# Clinical Efficacy of a Salicylic Acid–Containing Gel on Acne Management and Skin Barrier Function: A 21‐Day Prospective Study

**DOI:** 10.1111/jocd.70353

**Published:** 2025-07-19

**Authors:** Ye Liu, Yanjun Dan, Jiahong Yang, Xiaofeng He, Jingjing Liu, Yi Yi, Xing Chen, Xue Yin, Weina Song, Yueiqng Niu, Yijie Zheng, Yunfei Ai

**Affiliations:** ^1^ Department of Dermatology Huashan Hospital Affiliated to Fudan University Shanghai China; ^2^ L'Oréal Dermatological Beauty China, CeraVe Shanghai China; ^3^ L'Oréal China Research and Innovation Center Shanghai China

**Keywords:** acne management, clinical trial, salicylic acid, sebum reduction, skin barrier function

## Abstract

**Background:**

Acne vulgaris is a common skin condition characterized by excessive sebum production, inflammation, and compromised skin barrier function. Effective treatments should target both lesion reduction and skin hydration while maintaining tolerability.

**Objective:**

This study aimed to evaluate the efficacy and tolerability of a salicylic acid–containing gel in reducing sebum levels, improving skin hydration, strengthening the skin barrier, and alleviating acne severity over 21 days.

**Methods:**

A single‐center, prospective clinical trial was conducted on 42 participants (37 females, 5 males, mean age: 25.86 ± 6.69 years) with mild‐to‐moderate acne (IGA grade 2–3) and oily or combination skin. Participants applied the gel twice daily for 21 days. Sebum levels (Sebumeter SM815), trans‐epidermal water loss (TEWL, Tewameter TM Hex), and skin hydration (Corneometer CM825) were measured at multiple time points. Acne severity was assessed using the IGA scale, and self‐reported satisfaction surveys were collected.

**Results:**

Following the follow‐up period, sebum levels decreased by 23.65% (*p* < 0.05), while skin hydration increased by 40.5% (*p* < 0.05). TEWL decreased by 49.26% (*p* < 0.05), indicating enhanced skin barrier function. The IGA score improved by 23.81% (*p* < 0.001), demonstrating a significant reduction in acne severity. All participants (100%) reported satisfaction with the product, with notable improvements in oil control, acne reduction, and skin texture. The gel was well tolerated, with only 5% of participants reporting mild, transient itching, which resolved without intervention.

**Conclusion:**

The salicylic acid–containing gel effectively reduces acne lesions, regulates sebum production, enhances skin hydration, and strengthens the skin barrier, making it a suitable option for acne‐prone and sensitive skin. These findings suggest that the gel provides a well‐balanced approach to acne treatment by addressing both clinical efficacy and user comfort.

## 1 Introduction

Acne vulgaris (AV) is one of the most common dermatological conditions, affecting an estimated 9.4% of the global population, involving adults, adolescents, and preadolescents aged 9 years or older. AV is commonly characterized by an interplay of factors, including excessive sebum production, follicular hyperkeratinization, microbial colonization, and inflammation. AV significantly impacts quality of life, with psychosocial effects such as increased rates of anxiety, depression, and suicidal ideation [1, 2]. Effective treatment remains both a priority and a challenge in dermatology, particularly in individuals with sensitive or easily irritated skin [1, 3, 4]. Despite continuous advancements in acne treatment medications, evolving societal development and increased awareness have highlighted unmet needs in daily skincare. This presents an ongoing challenge for the development of skincare products that are both effective and suitable for daily use. Salicylic acid, a beta‐hydroxy acid (BHA), has been widely used in acne management due to its ability to penetrate sebum‐laden follicles, dissolve keratinized debris, and reduce comedogenic activity [5, 6, 7]. Additionally, salicylic acid exerts mild anti‐inflammatory properties, making it suitable for treating inflammatory lesions such as papules and pustules. Despite its proven efficacy, salicylic acid formulations can occasionally cause dryness or irritation, necessitating careful formulation to enhance tolerability [8, 9, 10].

Compromised skin barrier function, which is often observed in individuals with acne‐prone skin, presents significant challenges in the management of acne. The skin barrier is essential for maintaining hydration and protecting against environmental aggressors, but its dysfunction leads to increased trans‐epidermal water loss (TEWL), a hallmark indicator of barrier damage. Elevated TEWL is frequently noted in acne‐affected skin and contributes to heightened sensitivity, inflammation, and the progression of acne symptoms [11, 12]. Effective acne treatments need to address these interconnected issues by targeting not only the direct causes of acne but also the underlying barrier dysfunction. However, conventional treatments, while reducing lesions, often exacerbate dryness and irritation, making them unsuitable for long‐term use or for individuals with sensitive skin. An effective approach to acne management must consider the key underlying factors, including excessive sebum production, inflammation, and follicular keratinization. Simultaneously, it should support skin hydration and repair the damaged barrier to improve overall skin health [13]. By addressing these dual objectives, treatments can provide effective and sustainable outcomes for individuals managing acne.

Our study aims to evaluate the efficacy of a salicylic acid–containing gel designed to balance acne management with hydration and barrier repair. By integrating 2% salicylic acid with hydrating and skin‐soothing components, the gel aims to reduce sebum production, improve hydration, and diminish both inflammatory and non‐inflammatory lesions without inducing irritation. The study also explores immediate and cumulative effects over a 21‐day period, emphasizing both clinical and user‐reported outcomes.

## 2 Materials and Methods

### 2.1 Study Design

This was a single‐center, prospective clinical trial conducted over 21 days to evaluate the efficacy and tolerability of a salicylic acid–containing gel in improving acne‐related parameters and enhancing skin barrier function. The study adhered to the CONSORT guidelines, ensuring transparency and reproducibility. Ethical approval was obtained from the relevant institutional review board, and all participants provided written informed consent before enrollment. The trial was designed to assess both short‐term and cumulative effects of the gel. Immediate effects were evaluated post‐application on Day 0, and longer‐term outcomes were assessed on Days 7, 14, and 21. All assessments were conducted in a controlled environment to minimize variability due to external factors. Temperature (21°C ± 1°C) and humidity (50% ± 5%) were maintained in the testing rooms. Participants acclimatized for 30 min before measurements were taken to ensure stable skin conditions. This design allowed for a thorough examination of the gel's efficacy across multiple time points.

### 2.2 Participants

Participants were recruited through advertisements at dermatology clinics and social media platforms, with eligibility determined through initial screenings by dermatologists. Inclusion criteria required participants to be aged 18–44 years, have mild‐to‐moderate acne vulgaris (Investigator's Global Assessment [IGA] Grades 2–3), present with at least 5 inflammatory lesions (papules, pustules) and 10 non‐inflammatory lesions (open or closed comedones) on the face, and have an oily or combination skin type as determined by a dermatologist. Exclusion criteria included severe acne (nodulocystic or conglobata), active dermatological conditions such as eczema, psoriasis, or rosacea, use of systemic acne treatments within 12 weeks or topical treatments within 4 weeks before enrollment, known allergies to salicylic acid or any component of the gel formulation, pregnancy, breastfeeding, or plans to conceive during the study period, and participation in another clinical trial within the past 3 months. Eligible participants meeting these criteria were invited to participate, and their demographic details were recorded during baseline assessments.

### 2.3 Intervention

Participants were instructed to apply the salicylic acid–containing gel (2%) twice daily as part of their skincare routine. The formulation contained 2% salicylic acid as the primary active ingredient, along with 2.9% glycolic acid, 2.5% lactic acid, niacinamide, and three types of ceramides (EOP, NP, and AP), aiming to enhance exfoliation, improve skin tone, and support barrier repair. In the morning, participants cleansed their face using a mild, non‐medicated cleanser, patted it dry, and applied a thin layer of the gel to acne‐prone areas, avoiding the eye area. Sunscreen was then applied after the gel to protect against UV damage. In the evening, after cleansing, participants applied the gel in the same manner, ensuring no additional treatments, moisturizers, or cosmetics were applied immediately afterward. Participants were advised to avoid using any other acne treatments, exfoliants, or harsh skincare products during the trial. Compliance was monitored through self‐reported logs and periodic checks during follow‐up visits.

### 2.4 Evaluation of Efficacy and Skin Barrier Function of Salicylic Acid–Containing Gel Using

The study used a combination of instrumental measurements, dermatologist evaluations, and participant‐reported outcomes to comprehensively assess the efficacy and tolerability of the salicylic acid–containing gel. Sebum production was measured on the forehead using the Sebumeter SM815, which quantifies surface sebum levels in μg/cm^2^. Triplicate readings were obtained at each time point to ensure accuracy, and the average value was recorded. TEWL, a key indicator of skin barrier integrity, was measured on the forearm using the Tewameter TM Hex, which calculates water vapor loss (g/h·m^2^). Immediate post‐application effects of the gel on TEWL were evaluated at 3 and 6 h after the initial application. Skin hydration, reflecting the moisture content of the stratum corneum, was measured on the forearm using the Corneometer CM825, with results expressed in arbitrary capacitance units. In addition to these objective measures, participants completed structured questionnaires at baseline and at each follow‐up visit. These questionnaires used a 5‐point Likert scale to assess their perceived improvements in acne severity (IGA scale), skin texture, and hydration, as well as the gel's comfort during application and any adverse reactions. Open‐ended responses were also collected to provide qualitative insights into participant experiences with the product.

### 2.5 Safety Monitoring

Throughout the study, participants were encouraged to report any adverse events, including irritation, redness, dryness, or allergic reactions. Dermatologists conducted safety assessments at each visit to identify any potential issues. Adverse events were recorded and analyzed for severity and causality.

### 2.6 Statistical Analysis

Data were analyzed using SPSS 28.0 to evaluate the efficacy and tolerability of the salicylic acid–containing gel. Continuous variables, such as sebum production, TEWL, and hydration, were assessed at baseline and follow‐up visits using paired *t*‐tests for normally distributed data. For nonparametric data, the Wilcoxon signed‐rank test was employed to account for deviations from normality. Changes in lesion counts, including both inflammatory and non‐inflammatory lesions, were analyzed for statistical significance using paired tests. A significance threshold of *p* < 0.05 was applied to all comparisons. Descriptive statistics, including means, standard deviations (SD), medians, and ranges, were used to summarize the results, ensuring a clear presentation of the data. This approach provided robust analysis to detect meaningful changes over the 21‐day study period.

## 3 Results

### 3.1 Study Participants

A total of 42 participants (37 females, 5 males, mean age: 25.86 ± 6.69 years) completed the study. 81% of participants (34 individuals) reported having sensitive skin. All participants were healthy, with mild‐to‐moderate acne vulgaris (IGA Grades 2–3) and oily or combination skin types. The participants applied the salicylic acid–containing gel (2%) twice daily over a period of 21 days. The efficacy was assessed using a combination of instrumental measurements, dermatologist evaluations, and self‐reported satisfaction.

### 3.2 Evaluation of Skin Barrier Function of Salicylic Acid–Containing Gel Using

**FIGURE 1:**
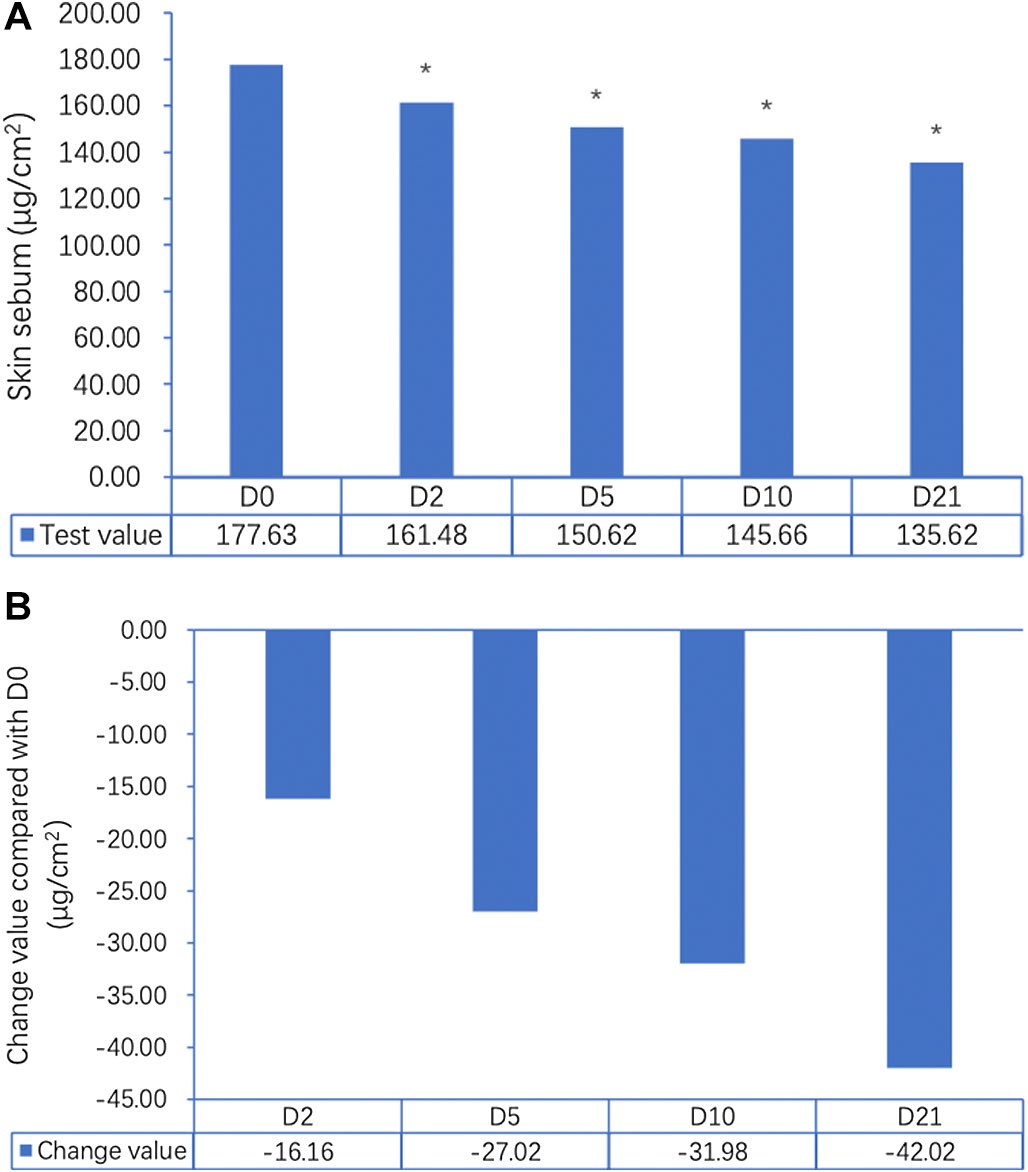
(A) Changes in skin sebum levels (µg/cm^²^) over time measured using the Sebumeter SM815 on the forehead at baseline (Day 0), Day 2, Day 5, Day 10, and Day 21. A significant reduction in sebum production was observed starting from Day 2 (161.48 µg/cm^²^), with continued decreases on Day 5 (150.62 µg/cm^²^), Day 10 (145.66 µg/cm^²^), and Day 21 (135.62 µg/cm^²^) compared to baseline (177.63 µg/cm^²^) (*p < 0.05 for all time points). (B) Mean change in sebum levels (µg/cm^²^) from baseline (D0) at each time point. Sebum levels decreased by −16.16 µg/cm^²^ at Day 2, −27.02 µg/cm^²^ at Day 5, −31.98 µg/cm^²^ at Day 10, and −42.02 µg/cm^²^ at Day 21, demonstrating the gel’s cumulative efficacy in reducing sebum secretion over 21 days.

**FIGURE 2:**
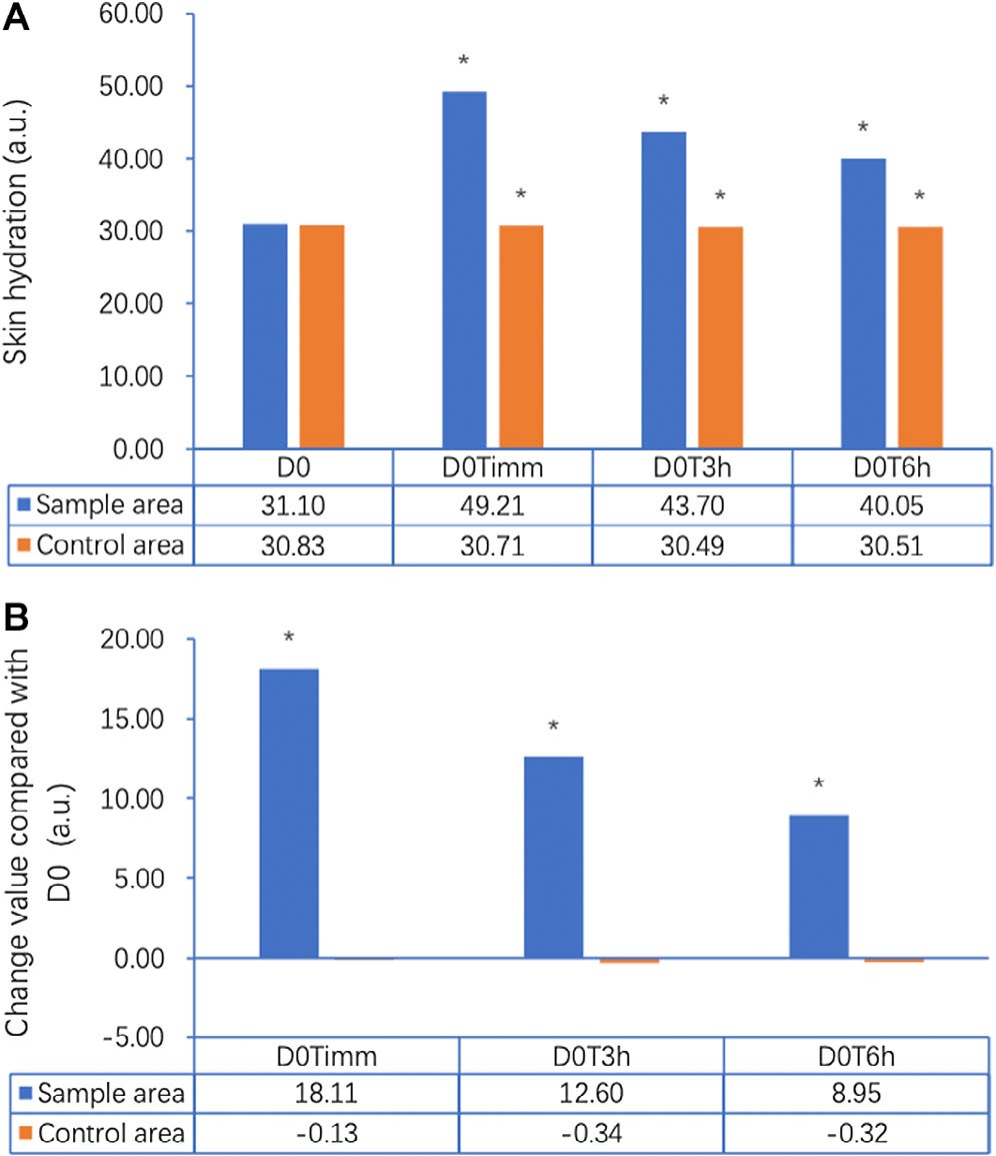
(A) Changes in skin hydration (a.u.) over time in the sample area and control area, as measured using the Corneometer CM825. Skin hydration in the sample area increased significantly from baseline (D0: 31.10 a.u.) to immediately after application (D0T1min: 49.21 a.u.), and remained elevated at 3 h (D0T3h: 43.70 a.u.) and 6 h (D0T6h: 40.05 a.u.) post‐application (*p < 0.05 for all time points). In contrast, hydration levels in the control area remained unchanged. (B) Change in hydration values (a.u.) compared with baseline (D0) for both the sample and control areas. The sample area showed a significant increase of 18.11 a.u. at 1 min, 12.60 a.u. at 3 h, and 8.95 a.u. at 6 h after application (*p < 0.05), whereas the control area exhibited negligible change at all time points. These results demonstrate the gel’s rapid and sustained moisturizing effect.

**FIGURE 3:**
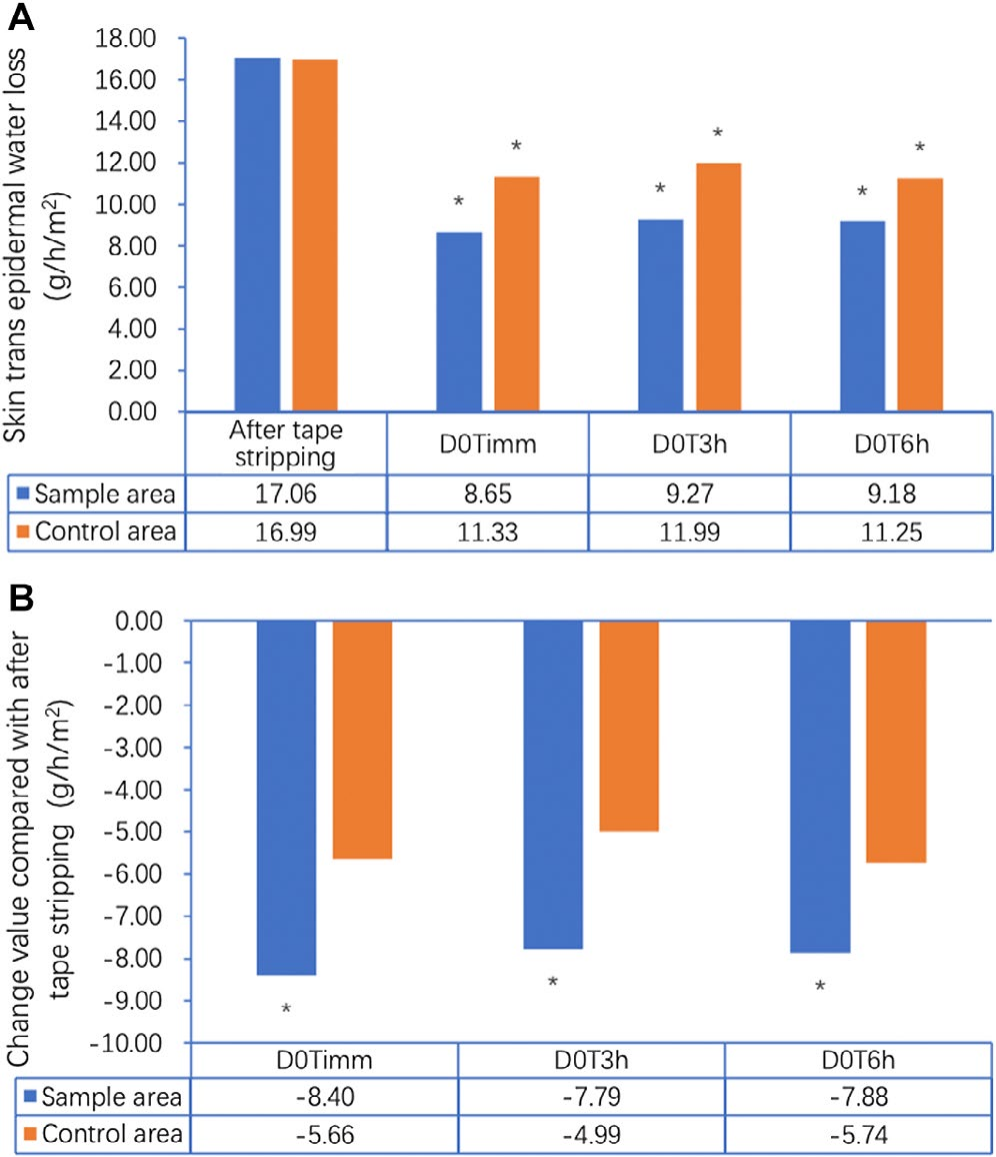
(A) Changes in skin trans‐epidermal water loss (TEWL, g/h/m^²^) over time in the sample and control areas following tape stripping, measured using the Tewameter TM Hex. TEWL significantly decreased in the sample area immediately after gel application (D0T1min: 8.65 g/h/m^²^), and remained low at 3 h (9.27 g/h/m^²^) and 6 h (9.18 g/h/m^²^) post‐application compared to post‐stripping levels (17.06 g/h/m²) (*p < 0.05). In contrast, the control area showed smaller reductions in TEWL. (B) Change in TEWL (g/h/m²) from post‐stripping baseline in both areas. The sample area demonstrated significant reductions of −8.40, −7.79, and −7.88 g/h/m^²^ at D0T1min, D0T3h, and D0T6h, respectively, while the control area showed more modest decreases. These results indicate that the salicylic acid–containing gel effectively improved skin barrier function shortly after application.

Sebum levels measured on the forehead using the Sebumeter SM815 demonstrated a statistically significant reduction over the 21‐day study period (*p* < 0.05) (Figure 1A,B). At baseline (Day 0), the average sebum level was 177.63 μg/cm^2^. By Day 2, there was an initial reduction to 161.48 μg/cm^2^ (↓9.10%, *p* < 0.05), followed by a further decrease to 150.62 μg/cm^2^ on Day 5 (↓15.21%, *p* < 0.05). By Day 10, sebum levels dropped to 145.66 μg/cm^2^ (↓18.00%, *p* < 0.05), and by Day 21, the final measurement recorded was 135.62 μg/cm^2^, reflecting a 23.65% total reduction from baseline (*p* < 0.05). This progressive decline suggests that continued application of the gel contributes to sustained oil control, which is crucial for acne‐prone skin. Skin hydration was evaluated using the Corneometer CM825, measuring changes in the moisture content of the stratum corneum. An immediate post‐application effect was observed, with hydration increasing by 18.11 a.u. at D0Timm (immediately), representing a significant improvement over baseline hydration levels (31.10 a.u.). This increase persisted over the next few hours, with hydration levels at D0T3h (43.70 a.u.) and D0T6h (40.05 a.u.), demonstrating continued moisture retention in the skin. By Day 21, hydration had improved by 40.5% compared to baseline (*p* < 0.05), confirming the gel's effectiveness in maintaining skin moisture levels over an extended period (Figure 2A,B). Notably, these hydration benefits contrast with many conventional acne treatments, which often lead to dryness and irritation. TEWL was assessed using the Tewameter TM Hex, measuring the amount of water vapor lost through the skin barrier. The results indicated that the gel application contributed to a statistically significant improvement in skin barrier function. Immediate post‐application (D0Timm) results showed a 45.67% reduction in TEWL compared to baseline. The effect was further sustained at D0T3h (↓46.19%) and D0T6h (↓49.26%), suggesting a continued strengthening of the skin barrier (Figure 3A,B). These reductions are clinically meaningful, as TEWL is a critical marker of barrier integrity, and acne‐prone skin is often characterized by increased water loss and impaired barrier function. The observed stabilization of TEWL levels throughout the study suggests that the gel not only treats acne but also enhances overall skin resilience by improving moisture retention and reducing susceptibility to external irritants.

### 3.3 Evaluation of Efficacy of Salicylic Acid–Containing Gel Using

**FIGURE 4:**
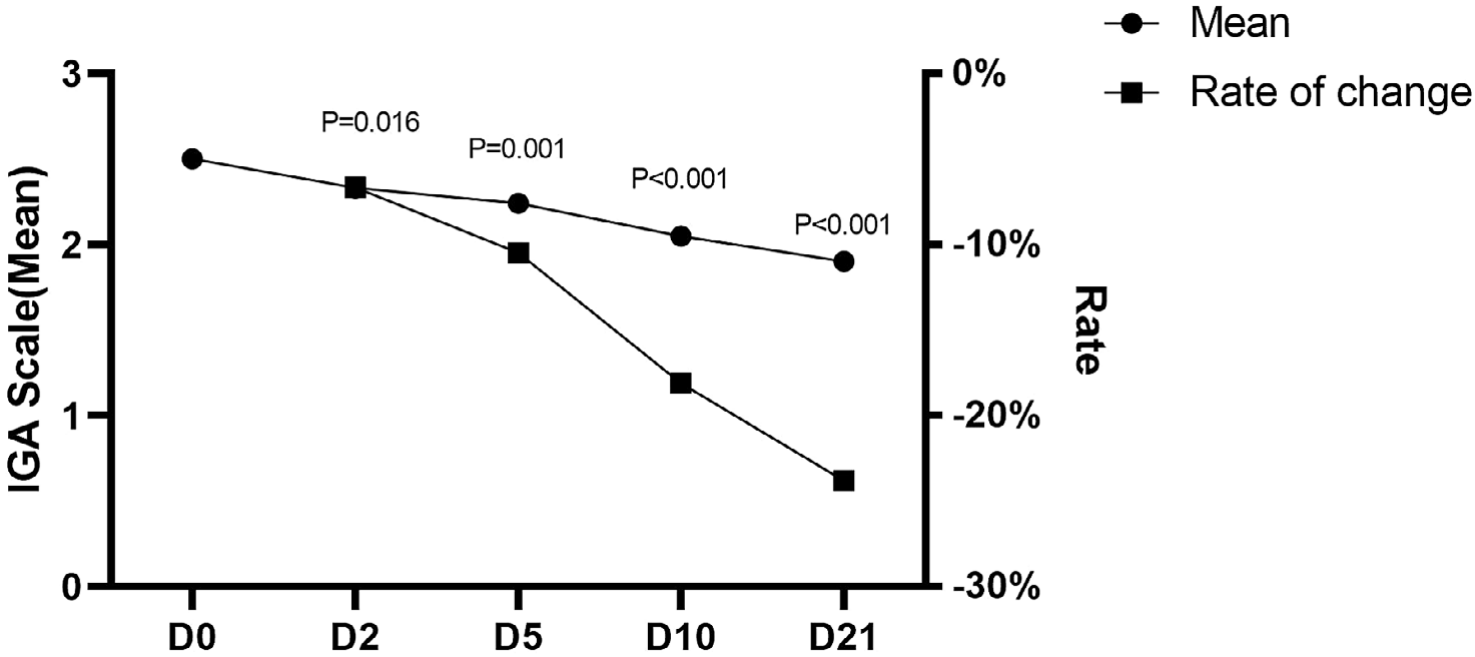
The improvement of Investigator's Global Assessment (IGA) scale over time. The improvement in the IGA scale, an indicator of acne severity, over 21 days with the salicylic acid–containing gel. The mean IGA score showed a significant reduction from 2.50 at D0 (baseline) to 1.90 by Day 21 (*p* < 0.001), indicating a 23.81% improvement. The rate of change in the IGA score further supported these findings, with a 6.67% decrease by Day 2, 10.48% by Day 5, 18.10% by Day 10, and a cumulative 23.81% improvement by Day 21 (*p* < 0.001). These results highlight the gel's effectiveness in reducing acne severity over the study period.

The IGA scale was used to track changes in acne severity over the study period (Figure 4). At baseline (Day 0), the mean IGA score was 2.50, indicative of mild‐to‐moderate acne severity. By Day 2, the IGA score had improved by 6.67%, demonstrating early treatment effects. By Day 5, there was a 10.48% reduction, followed by an 18.10% reduction by Day 10. By Day 21, the IGA score had significantly improved to 1.90 (↓23.81%, *p* < 0.001), confirming a clinically relevant reduction in acne severity. These results support the gel's ability to effectively manage both inflammatory and non‐inflammatory acne lesions without causing irritation or rebound effects.

### 3.4 Self‐Assessment Surveys

Participants reported high satisfaction rates, reinforcing the instrumental findings (Table 1). By Day 21, 100% of participants found the product easy to apply and comfortable for daily use. 95% reported that the gel effectively controlled oiliness. 92% noticed visible improvements in acne severity. 87% observed an increase in skin hydration. 90% stated that their skin felt healthier and smoother after continuous application. Additionally, self‐reported improvements in pore size, radiance, and overall skin appearance were consistently observed across time points.

**TABLE 1:** Self‐reported improvements in skin condition.

Question	Satisfaction			
	D2	D5	D10	D21
Product is easy to apply	100%	100%	100%	100%
Product absorbs quickly	88%	98%	98%	98%
Product feels pleasant at application	86%	98%	100%	100%
Product feels light weight on skin	93%	98%	98%	98%
Product is NOT sticky on skin	95%	98%	98%	98%
Product does NOT feel oily greasy on skin	95%	100%	100%	100%
Skin feels soothed calmed	86%	95%	100%	100%
Skin feels comforted	88%	100%	100%	100%
Feels refined	90%	100%	100%	100%
Skin feels soft	88%	100%	100%	100%
Skin feels less oily	79%	95%	100%	100%
Skin feels balanced (not too oily not too dry)	83%	98%	100%	100%
Visible pore appears unclogged	93%	98%	100%	100%
Visible pore appears smaller	86%	98%	100%	100%
Visible acne blemishes look reduced	83%	98%	100%	100%
No new visible imperfection since I started using this product	88%	100%	100%	100%
Skin texture looks smoother	88%	98%	100%	100%
Skin looks healthier	90%	100%	100%	100%
Skin tone looks more even	93%	100%	100%	100%
Skin looks more radiant	86%	100%	100%	100%
Overall skin appearance looks more improved	88%	100%	100%	100%
Product is suitable for my skin type	/	/	/	100%
Overall, how satisfied are you with the product?	/	/	/	100%

*Note:* By Day 21, 100% of participants reported satisfaction with the product, indicating its high acceptability. Notable improvements were observed in skin texture, oiliness control, pore visibility, acne blemish reduction, and overall skin appearance, with near‐total agreement by Day 10 and complete satisfaction by Day 21. These findings highlight the gel's rapid and sustained benefits in enhancing skin condition while ensuring a pleasant user experience.

### 3.5 Safety and Tolerability

The salicylic acid–containing gel was well tolerated throughout the study. Dermatologist evaluations revealed no significant adverse events, such as erythema, dryness, or peeling. Minor itching was reported by 5% of participants during the first week but resolved without intervention. No participants discontinued the gel due to adverse reactions, confirming its safety and tolerability for the duration of the study.

## 4 Discussion

Our results show that the salicylic acid–containing gel effectively reduces sebum production, improves skin hydration, enhances skin barrier function, and reduces acne severity over a 21‐day period. The progressive 23.65% reduction in sebum levels by Day 21 supports the gel's ability to regulate oil production, an important factor in acne management. The early sebum reduction (9.10% by Day 2) suggests that the gel begins working soon after application, which could help users see noticeable improvements early in treatment. In addition to controlling oil production, the gel had a clear impact on skin hydration. Many acne treatments, including benzoyl peroxide and retinoids, are associated with dryness and irritation, making it difficult for individuals with sensitive skin to maintain treatment. However, this gel significantly improved hydration levels, with an immediate post‐application increase of 58.23% and a 40.5% improvement by Day 21. The ability to increase hydration while managing acne suggests that the gel has moisturizing properties, making it more tolerable for individuals prone to dryness or sensitivity.

The study also showed improvements in skin barrier function, measured by TEWL. Within 6 h post‐application, TEWL was reduced by 49.26%, suggesting that the gel helps strengthen the skin's natural protective barrier. Acne‐prone skin is often more prone to moisture loss, and compromised barrier function can lead to irritation and increased sensitivity. The ability of this gel to maintain hydration and reduce TEWL indicates that it may support overall skin health rather than just addressing acne symptoms. Acne severity, measured by the IGA scale, also showed a 23.81% reduction by Day 21. The improvement in IGA scores was observed as early as Day 2 (6.67%), with continued reduction by Day 10 (18.10%). This steady decline in acne severity suggests that the gel effectively targets both inflammatory and non‐inflammatory acne lesions over time.

Our findings are consistent with previous studies demonstrating the efficacy and safety of salicylic acid–containing skincare products in managing mild‐to‐moderate acne. A large multicenter trial showed that 2% supramolecular salicylic acid was comparable to adapalene in reducing acne lesions, with good tolerability and improvements in hydration and barrier function. Another randomized study confirmed that a serum and mask containing salicylic acid and lipohydroxy acid improved comedones, post‐inflammatory erythema, and pigmentation, while enhancing skin hydration and reducing sebum [14, 15]. These results support our observations of significant reductions in sebum and TEWL, alongside improved hydration and acne severity over a short 21‐day period, highlighting the benefit of barrier‐supportive formulations for sensitive, acne‐prone skin.

From a user experience perspective, participant‐reported satisfaction was high. By Day 21, 100% of participants were satisfied with the product, and 95% noted effective oil control, while 92% saw visible acne improvement. The gel was also well tolerated, with only 5% of participants reporting mild itching, which resolved without requiring additional treatment. These findings indicate that the gel is suitable for daily use, even for individuals with sensitive skin. Overall, the study demonstrates that this salicylic acid–containing gel is an effective acne treatment that not only reduces acne lesions but also improves hydration and barrier function. Given the high user satisfaction and minimal side effects, it may be a suitable option for long‐term acne management, particularly for those with sensitive or irritation‐prone skin. Further studies with longer follow‐up periods could help determine if continued use provides even greater benefits in skin quality and acne control, and future studies with ≥ 28‐day follow‐up are needed to assess sustained effects on barrier repair and acne recurrence.

## 5 Conclusions

The salicylic acid–containing gel demonstrated strong efficacy in reducing sebum levels, improving skin hydration, enhancing skin barrier function, and reducing acne severity over 21 days. The progressive reduction in sebum production and significant hydration improvements indicate that the gel effectively manages acne while maintaining skin moisture, addressing a common concern in acne treatment. The 49.26% reduction in TEWL suggests that the gel also strengthens the skin's natural barrier, making it a potentially beneficial option for individuals with acne‐prone and sensitive skin. The 23.81% improvement in the IGA score confirms its ability to reduce both inflammatory and non‐inflammatory acne lesions, with visible results starting as early as Day 2. Self‐reported satisfaction was 100% by Day 21, highlighting the gel's acceptability and ease of use. The absence of significant adverse effects further supports its suitability for long‐term use. Given its efficacy, tolerability, and dual action in acne treatment and skin hydration, this gel could serve as a comprehensive solution for acne‐prone individuals, particularly those seeking gentle yet effective care. Future studies with longer follow‐up periods may further confirm its long‐term benefits and sustained acne management potential.

## Author Contributions

Liu and Yanjun Dan contributed equally to this work as co‐first authors. Ye Liu and Yanjun Dan were responsible for the conceptualization and design of the study. Jiahong Yang, Xing Chen, Xue Yin, Weina Song, Yueqing Niu, and Yijie Zheng conducted the experiments and collected the data. Xiaofeng He, Jingjing Liu, and Yi Yi contributed to data analysis, interpretation, and manuscript drafting. Yunfei Ai supervised the study, provided critical revisions, and approved the final manuscript. All authors reviewed and approved the final version of the manuscript.

## Ethics Statement

This study was approved by the SGS Ethics Committee for Clinical Research.

## Consent

Obtaining written informed consent from all participants.

## Conflicts of Interest

The authors declare no conflicts of interest.

## Data Availability

The data that support the findings of this study are available from the corresponding author upon reasonable request.
