# Correction to “Clinical Efficacy of a Salicylic Acid–Containing Gel on Acne Management and Skin Barrier Function: A 21‐Day Prospective Study”

**DOI:** 10.1111/jocd.70589

**Published:** 2025-12-05

Liu Y, Dan Y, Yang J, et al. “Clinical Efficacy of a Salicylic Acid‐Containing Gel on Acne Management and Skin Barrier Function: A 21‐Day Prospective Study.” *J Cosmet Dermatol* 2025;24(7):e70353.

Author name correction:

The name of one author was misspelled as “Yueiqng Niu” in the byline and author information section. The correct spelling should be “Yueqing Niu.”

Author affiliation correction:

The affiliation of the abovementioned author was incorrectly listed as “2. L'Oréal Dermatological Beauty China, CeraVe, Shanghai, China.” Her correct affiliation is “3. L'Oréal China Research and Innovation Center, Shanghai, China.”

We apologize for this error.

